# Clinical Characteristics and Outcomes in Multisystemic Inflammatory Syndrome in Children (MIS-C) Associated with COVID-19: A 12-Month Prospective Study

**DOI:** 10.3390/microorganisms13061405

**Published:** 2025-06-16

**Authors:** Viorela Gabriela Nitescu, Diana-Andreea Usurelu, Teodora Olsavszky, Ana-Maria Mihalcea, Andra Postelnicu, Ruxandra Florea, Simona Stanca, Iolanda Cristina Vivisenco, Madalina Elena Petran, Maria-Dorina Craciun, Carmen-Daniela Chivu, Alexandru Ulici, Coriolan Emil Ulmeanu

**Affiliations:** 1 Discipline of Pediatrics, Faculty of Dentistry, Carol Davila University of Medicine and Pharmacy, 010221 Bucharest, Romania; diana-andreea.usurelu@drd.umfcd.ro (D.-A.U.); maria.craciun@umfcd.ro (M.-D.C.);; 2 Department of Pediatrics, Grigore Alexandrescu Clinical Emergency Hospital for Children, 011743 Bucharest, Romania; 3 Department of Infection Prevention and Control, Grigore Alexandrescu Clinical Emergency Hospital for Children, 011743 Bucharest, Romania; 4 Department of Pediatric Orthopedics, Grigore Alexandrescu Clinical Emergency Hospital for Children, 011743 Bucharest, Romania

**Keywords:** cardiac dysfunction, children, coronary aneurysms, COVID-19, MIS-C, pulmonary sequelae

## Abstract

Multisystemic inflammatory syndrome in children (MIS-C) is a rare but potentially severe condition that affects multiple organ systems. This study aimed to assess the clinical characteristics and outcomes of patients diagnosed with multisystemic inflammatory syndrome in children (MIS-C) associated with COVID-19. A 12-month prospective study was conducted at the “Grigore Alexandrescu” Clinical Emergency Hospital for Children, Bucharest. This study included children aged 0–18 years who were diagnosed with MIS-C, as defined by the World Health Organization (WHO), the Royal College of Paediatrics and Child Health (RCPCH), and the Centers for Disease Control and Prevention (CDC) criteria. Data on age, gender, clinical and laboratory findings, treatment, and outcomes were analyzed. Follow-up evaluations occurred at one, three, six, nine, and twelve months post-discharge. Among 36 patients (47.3% female, 52.7% male; mean age, 9.9 years), fever and inflammatory syndrome were present in all patients. Other common symptoms included mucocutaneous (63.8%), gastrointestinal (52.7%), cardiac (47.2%), pulmonary (38.8%), and neurological (11.1%) manifestations. At admission, 14/36 were IgM-positive, while 34/36 were IgG-positive. Follow-up revealed sequelae in two patients, including coronary aneurysms and ground-glass pulmonary opacities. Although MIS-C can be severe, most patients had favorable outcomes with proper treatment. Few long-term, organ-specific complications were observed, highlighting the importance of systematic monitoring to ensure full recovery.

## 1. Introduction

In 2020, a few months after the start of the Coronavirus disease 2019 (COVID-19) pandemic, an increase in hospitalizations of children with clinical features resembling toxic shock syndrome or Kawasaki disease was reported [1,2,3]. This condition, defined as multisystemic inflammatory syndrome in children (MIS-C), was characterized by main Health Organizations as a severe hyperinflammatory condition with fever and multiorgan involvement that develops 2 to 8 weeks following confirmation of severe acute respiratory syndrome coronavirus 2 (SARS-CoV-2) infection through positive reverse transcriptase polymerase chain reaction (RT-PCR) or antigen test [1,2,3]. The pathogenesis of MIS-C is thought to involve a delayed, dysregulated immune response. Elevated cytokine levels have been observed, leading to endothelial injury, capillary leak, and multiorgan dysfunction, supporting its classification as a post-infectious inflammatory syndrome [4].

Although the acute phase of MIS-C has been thoroughly described, its short- and long-term sequelae are still not well understood [4,5]. Likewise, knowledge about rehabilitation needs following hospital discharge remains limited. Additional data are required to establish a structured follow-up. In the current study, we aimed to analyze the epidemiological, clinical, and paraclinical characteristics of children diagnosed with MIS-C and their outcomes over a 12-month follow-up period.

## 2. Materials and Methods

### 2.1. Study Design

This prospective observational study was conducted in Bucharest, Romania, at the “Grigore Alexandrescu” Clinical Emergency Hospital for Children, which is a tertiary care hospital that had a dedicated unit for COVID-19 cases during the pandemic. All patients aged ≤18 years who were admitted to the hospital with MIS-C between October 2020 and November 2022 and met the MIS-C case definition criteria: children aged 0–18 years old with fever lasting more than 3 days, with elevated inflammatory markers, multisystemic involvement (at least two systems), no other cause of inflammation, and evidence of SARS-CoV-2 infection or likely contact [3].

This study was performed in accordance with the principles of the Declaration of Helsinki. Approval was granted by the Ethics Committee of “Grigore Alexandrescu” Clinical Emergency Hospital for Children (Date: 15 October 2020, No/10105).

All parents signed an informed consent form regarding the use of anonymous data. Parents were informed about the clinical procedures, laboratory testing, and examinations to be undertaken during follow-up hospital visits.

### 2.2. Patient Evaluation Methodology

**Figure 1:**
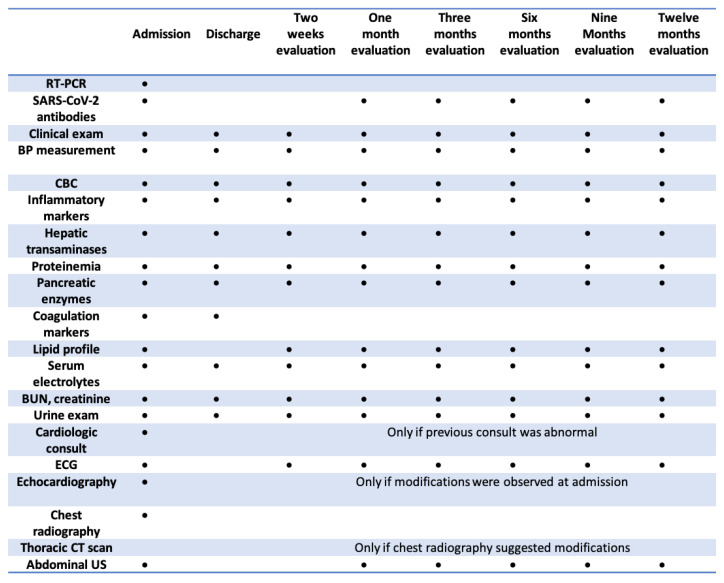
Patient evaluation protocol.

All patients underwent RT-PCR tests for SARS-CoV-2 RNA detection upon admission, and serological test results for previous SARS-CoV-2 exposure (IgM and IgG antibodies) were obtained. Patients with sepsis or other causes of inflammation were excluded from this study. The initial admission was considered the start of the monitoring period. After discharge, patients underwent periodic follow-up visits at 2 weeks or one month after discharge, depending on the severity of the disease, and at three, six, nine, and twelve months after the first hospitalization. We utilized a well-defined monitoring protocol, as summarized in Figure 1. Dynamic SARS-CoV-2 serology (IgG antibodies) was monitored, and quantitative tests were performed at every follow-up visit until the results were negative. Hospital staff made the follow-up appointments. Children who failed to attend these periodic visits were considered lost to follow-up.

### 2.3. Data Collection

All data were collected from electronic medical records and hospital notes. These data included demographics, clinical signs and symptoms during the acute phase of MIS-C and at the follow-up assessment(s), medical history, laboratory and pathogen test results, pulmonary radiography and computed tomography (CT) imaging, electrocardiography (ECG), echocardiography (ECHO), abdominal ultrasound (US) supportive care, treatment modalities, and outcomes for all children who met the inclusion criteria.

### 2.4. Statistical Analysis

Continuous data are summarized as means and ranges (minimum–maximum), and nominal data are summarized as absolute counts and percentages (%). In cases of missing data, valid counts are shown as n/N (%). Descriptive statistics were used to present baseline characteristics. Categorical variables were compared using Fisher’s exact tests. Comparisons of continuous variables were analyzed using the *t*-test or the nonparametric Mann-Whitney U test. A *p*-value < 0.05 was considered to indicate statistical significance. Statistical analysis was performed using IBM-SPSS Statistics, version 29.0.2.0 (2023).

## 3. Results

### 3.1. Demographic, Epidemiological, and Clinical Characteristics

Our study included 36 previously healthy patients without comorbidities, 17 females and 19 males, with a mean age of 9.9 years (Table 1). Fifteen patients (41.6%) had a recently documented SARS-CoV-2 infection, with an average duration of 35.5 days (range 14–48) between the diagnosis of acute SARS-CoV-2 infection and MIS-C onset. Fourteen children were positive for IgM antibodies, and thirty-four children were positive for IgG antibodies and anti-nucleocapsids (Table 2). One patient had a positive SARS-CoV-2 RT-PCR test upon admission. No patient was vaccinated prior to admission or during the follow-up period.

**Figure 2:**
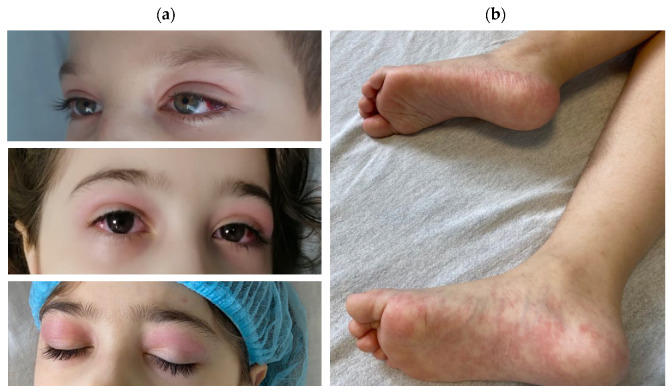
Mucocutaneous findings observed in hospitalized children with MIS-C: (**a**)subconjunctival hemorrhage, nonexudative conjunctivitis, and periorbital edema and (**b**) plantar erythema.

The clinical characteristics of patients with MIS-C are presented in Table 3. All patients presented with fever associated with mucocutaneous lesions (Figure 2), 23 patients with conjunctivitis, 23 with rash, 4 with lymphadenopathies, gastrointestinal symptoms (18 patients with abdominal pain, 16 with vomiting, and 14 with diarrhea), cardiovascular manifestations (8 patients with palpitations, 3 with tachycardia, 3 with bradycardia, and 5 with cardiac murmur), respiratory symptoms (8 patients with cough and 5 with dyspnea), or neurological manifestations (8 patients with headache and 6 with lethargy). Signs of shock were present in four patients (11.1%). The average hospitalization duration was 9 days (range 4–18).

### 3.2. Laboratory Investigations

Laboratory investigations, including complete blood counts, inflammatory markers, and organ function tests at admission and discharge, are detailed in Table 4. All patients had elevated inflammatory markers at admission according to the case definition, lymphopenia in 32 (88.89%) patients, neutrophilia in 30 (83.34%) patients, thrombocytopenia in 6 (16.67%) patients, anemia in 18 (50%) patients, liver cytolysis in 9 (25%) patients, coagulopathy in 12 (33.34%) patients. Triglycerides were elevated in 15 (41.67%) patients. Urinary protein excretion was evaluated quantitatively at admission in 19 patients (52%) with nephritic proteinuria (>0.3 g/24 h). Together, these findings show that patients with MIS-C had an elevated inflammatory profile with multiple organ dysfunction. No statistically significant differences were seen regarding age, gender, or days between the diagnosis of COVID-19 and the debut of MIS-C symptoms and laboratory parameters.

### 3.3. Cardiac Dysfunction Evaluation

Levels of cardiac dysfunction markers were elevated in 14 (38.8%) patients, with troponin I > 0.05 ng/mL in 2 (5.56%) patients, CK-MB > 100 pg/mL in 2 (5.56%) patients, brain natriuretic peptide (BNP) > 100 pg/mL in 10 (27.78%) patients with a mean value of 243.63 pg/mL (range 0.3–1210), myoglobin > 107 ng/mL in 5 (13.8%) patients, and D-dimer > 400 ng/mL in all our patients (100%).

Electrocardiograms were performed on all our patients, and the results showed tachycardia in six (16.6%) patients and bradycardia in two (5.5%) patients. One patient (2.77%) had arrhythmia (ventricular extrasystole with a left bundle branch block appearance) and ST-segment depression. Echocardiograms revealed systolic dysfunction of the left ventricle in nine (27.27%) patients, pericardial effusion in five (15.15%) patients, and coronary artery aneurysms in two (6.06%) patients with z scores > 2.5 (ACD z scores of 3.8 and 4) (Table 5).

### 3.4. Imaging Investigations

Chest X-rays were performed to better characterize pulmonary dysfunction. The findings revealed anomalies in 14 patients (47%), 7 with diffuse interstitial infiltrates, 6 with localized infiltrates, 1 with atelectasis, and 1 with pleural effusion. Additionally, CT revealed ground-glass opacities in two patients, pleural effusion in two patients, and pulmonary emphysema in one patient.

On abdominal ultrasound, 13 patients (43.33%) had liver enlargement, 20 (66.66%) had changes in liver echogenicity, 4 (13.33%) had splenomegaly, and 11 (36.66%) had renal changes, with 4 (13.33%) having pelvicalyceal dilation and 10 (33.33%) having corticomedullary differentiation loss. Fluid effusion was found in nine (30%) patients: two (6.66%) had pericardial effusion, three (10%) had pleural effusion, and five (16.66%) had abdominal fluid effusion. The imaging results are detailed in Table 6.

### 3.5. Treatment During Hospitalization and at Discharge

All 36 patients received intravenous corticosteroids (methylprednisolone), and 20 patients (55.5%) received in association with the steroids, treatment with intravenous human immunoglobulin. This association was reserved for severe cases. Twenty-three (63.8%) patients received deep vein thrombosis prophylaxis with LMWH enoxaparin. Thirty-five out of thirty-six patients (97.2%) required antibiotic therapy, and eighteen patients (50%) required local ophthalmologic treatment. Eight patients (22.2%) required diuretic therapy with furosemide and spironolactone. Four patients (11.1%) were admitted to the intensive care unit and received supportive inotropic therapy. Additionally, three patients (8.3%) required oxygen therapy for acute respiratory failure (SatO2 < 92%). One patient (2.7%) died due to severe respiratory and neurological complications after 47 days.

At discharge, all patients received treatment with 1 mg/kg oral methylprednisolone for two weeks or one month, depending on the severity of the initial disease, followed by a gradual tapering of doses.

### 3.6. Follow-Up Evaluations

After discharge, patients were initially re-evaluated at 2 weeks or one month, depending on the severity of MIS-C, with subsequent follow-up visits at 3, 6, 9, and 12 months after disease onset. However, 64% of patients did not come to the scheduled medical appointments due to low adherence or limited access to healthcare, because of reduced socio-economic resources.

**Figure 3:**
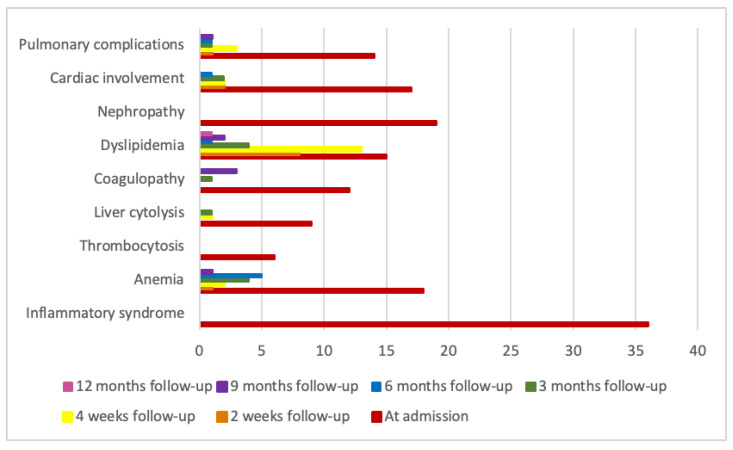
Clinical and biological complications at admission and during follow-up.

The medical parameters analyzed are detailed in Table 7, and a graphical illustration is provided in Figure 3.

**Figure 4:**
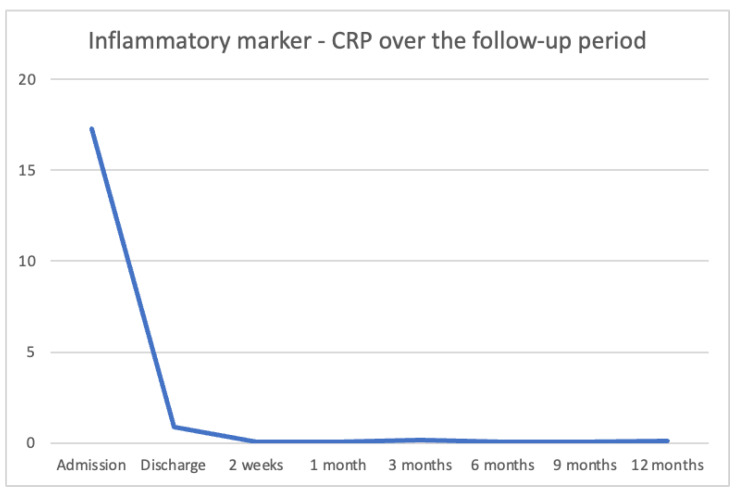
Inflammatory marker CRP over the months.

All inflammatory markers returned to normal levels after 14–30 days, with a median CRP of 0.08 mg/dL, as shown in Figure 4.

Left ventricular systolic function was normal in all patients except one with a coronary aneurysm and another with hypertrophic cardiomyopathy. Respiratory complications decreased. CT scans at one month showed ground-glass opacities in only four patients. Abdominal ultrasound revealed changes in liver echogenicity in 12 (38.6%) patients.

At the 3-month follow-up, 31 patients (88.5%) presented with elevated inflammatory markers. Laboratory blood tests revealed anemia in two (6.4%) patients. One patient presented with coagulopathy and liver cytolysis and was negative for viral markers. Echocardiograms showed one patient with a coronary aneurysm and another with hypertrophic cardiomyopathy. In particular, one patient developed type 2 diabetes and was referred to an endocrinologist.

Twenty patients returned for the 6-month follow-up. Patients were lost due to the lack of compliance and adherence to monitoring. Laboratory tests revealed anemia in five patients (25%), without inflammatory syndrome or other significant changes. Vitamin D3 deficiency was detected in four patients (20%). Echocardiography was within normal limits, without ventricular dysfunction, coronary aneurysms, or dilations. On abdominal ultrasound, two (10%) patients showed changes in hepatic echogenicity: one patient had linear micro-echogenicity, and one patient had a hyperechogenic liver.

At the 9-month re-evaluation, 12 patients (33.3%) returned for follow-up. Laboratory tests revealed mild anemia in one patient (8.3%), but none of the patients had inflammatory syndrome. Another three patients (25%) showed slight changes in coagulation time. No cardiac abnormalities were noted, and all abdominal ultrasounds performed were within normal limits.

Thirteen patients (36.11%) returned for the 12-month follow-up. One patient showed increased hepatic echogenicity, and one patient showed abnormal changes on chest X-ray, consisting of micronodular opacities inferior to the right hilum, consistent with previous investigations.

## 4. Discussion

This study analyzes the clinical characteristics of 36 patients admitted with MIS-C at our clinic, as well as the outcomes of 30% of patients who had come to the follow-up evaluation over a period of 12 months. The demographic characteristics of MIS-C patients were consistent with previous findings, with no significant impact of age or gender on clinical outcomes [4,5,6]. All patients had either confirmed SARS-CoV-2 exposure, a positive RT-PCR, or antibodies against spike or nucleocapsid proteins, supporting the link between MIS-C and SARS-CoV-2 infection [7,8].

The clinical presentation varied, aligning with prior reports [9,10,11,12,13,14,15,16], with fever universally present. The most common features in our cohort were mucocutaneous lesions (63.8%), gastrointestinal symptoms (52.7%), and cardiovascular involvement (47.2%). Dermatological findings ranged from mild rashes to generalized erythematous macules and papules, often with conjunctivitis [17] and lip or palmoplantar changes [18,19]. Gastrointestinal manifestations, the second most frequent symptom, support prior findings suggesting gut involvement or underlying inflammatory bowel disease [20,21,22].

Cardiac involvement was observed in nearly half of the patients. Only four developed cardiovascular collapse or cardiogenic shock, a lower incidence compared to reports of 40–80% [16,23,24]. ECG abnormalities were common, including one transient case of ventricular extrasystole with left bundle branch block (LBBB) and ST depression. Left ventricular systolic dysfunction occurred in 25%, while coronary aneurysms were rare (two cases) [25,26,27]. Limited imaging access may have underestimated cardiac findings; other studies suggest that CT and MRI detect more coronary and myocardial changes than echocardiography alone [28]. Other clinical manifestations included respiratory symptoms, such as cough or dyspnea, for which chest X-rays were performed, showing diffuse interstitial infiltrate, some with pleural effusion. CT scans evidenced ground-glass opacities in a small number of patients. These findings might suggest differences between acute COVID-19 episodes, severe respiratory distress, and lung damage, and the inflammatory mechanisms that characterize MIS-C [29,30,31].

All children had increased inflammatory markers, such as CRP, ESR, and ferritin, which have also been reported in other MIS-C cohorts across Europe and the U.S. [8,32,33,34]. The majority of our patients had lymphopenia, and some presented with neutrophilia, thrombocytopenia, and abnormal fibrinogen levels. Liver cytolysis, which was observed in 25% of our patients, was further investigated using abdominal ultrasound, which suggested, as published in other studies, the complexity of this syndrome and the variability of affected organs [35,36]. Coagulopathy, with higher levels of D-dimer, altered fibrinogen, increased prothrombin time, and increased INR, indicates a prothrombotic state, consistent with other studies [37,38]. Additionally, renal involvement was noted, the prevalence of which was slightly lower than that in other studies [39,40].

Patients with MIS-C can experience rapid deterioration and should, therefore, receive thorough monitoring and prompt therapeutic management [23]. Anti-inflammatory treatment, such as steroids, was a necessary treatment, and in more than half of the patients, it was associated with intravenous immunoglobulin (IVIg), which appeared to be effective. In our study population, the clinical course of the disease was favorable, with only one death, consistent with the findings of other studies [41,42,43,44].

Long-term complications associated with MIS-C were monitored during follow-up visits over a period of one year, which is similar to approaches used at other centers [10,44]. Despite the initial severity of the illness among our patients, few organ-specific sequelae were observed [45,46,47,48,49,50]. Among our patients, two had cardiac dysfunction, one had persistent coronary aneurysms and was referred to the pediatric cardiology unit, and the other presented with hypertrophic cardiomyopathy, which resolved 6 months after discharge. Another patient showed lung sequelae, consisting of micronodular opacities inferior to the right hilum, which persisted even 12 months after the first hospitalization. One patient developed type 2 diabetes 3 months after admission and was referred to an endocrinologist, receiving oral antidiabetic treatment. The majority of patients recovered after the first follow-up visit, with nearly all having no symptoms accompanied by normal laboratory findings. These findings align with other published studies, suggesting that the long-term complications associated with MIS-C are minimal [48,49,50,51,52].

The present study has several limitations. First, the number of patients included in this study was small, making comparisons among clinical forms difficult. Second, without a control group, this study does not provide evidence on the effectiveness of MIS-C treatment. Patients were treated with corticosteroids with or without IVIg, according to local protocols. Further studies are needed to determine the optimal treatment or whether other agents targeting specific inflammatory pathways or cells may be preferable. Third, only 36% of patients could come to the follow-up visits, due to low compliance or limited access to healthcare, to reduced socioeconomic resources.

It is important to emphasize that long-term multidisciplinary follow-up studies are needed since it is vital to determine whether affected patients experience chronic impairment or other sequelae. The information related to the clinical features and outcomes of children hospitalized with MIS-C in Romania that was obtained in our study is relevant to our understanding of the disease and its management, and the authors believe it to be an important contribution to the understanding of SARS-CoV-2 infection in children and its short- and long-term consequences.

## 5. Conclusions

In conclusion, patients with MIS-C were associated with favorable outcomes in the majority of cases. Despite the initially severe illness, with prompt therapeutic management and monitoring, patient outcomes were favorable, with a low mortality rate and good recovery. In our study, 36% of patients were evaluated over 12 months, being identified with a few organ-specific sequelae, notably coronary aneurysms and ground-glass pulmonary opacities. This study aims to expand the knowledge of this condition, and providers should take into consideration a long-term following period, to better understand and manage this disease.

## Figures and Tables

**Table 1:** Demographic characteristics of patients admitted to the hospital with MIS-C diagnosis.

Demographic Characteristics	Value
Patients, n	36
Age, years, median	9.9
Age range	8 months–17 years
Females, n (%)	17 (47.2)
Males, n (%)	19 (52.7)
City environment, n (%)	21 (58.3)
Rural environment, n (%)	15 (41.6)

**Table 2:** SARS-CoV-2 infection status.

SARS-CoV-2 Infection Status	Value
Documented COVID-19	15 (41.6)
Days between diagnostic of acute COVID-19 and MIS-C debut	35 (14–62)
Positive SARS-CoV-2 RT-PCR at admission n (%)	1 (2.7)
IgM at admission positive, n (%)	14 (38.8)
IgG at admission positive, n (%)	34 (94.4)

**Table 3:** Clinical characteristics of patients with MIS-C admitted to the hospital.

Clinical Characteristics	Value n (%)
Fever	36 (100)
Rash	23 (63.8)
Cough	8 (22.2)
Dyspnea	5 (13.8)
Palpitations	8 (22.2)
Bradycardia	3 (8.3)
Tachycardia	3 (8.3)
Cardiac murmur	5 (13.8)
Signs of shock	4 (11.1)
Abdominal pain	18 (50)
Nausea, vomiting	16 (44.4)
Diarrhea	14 (38.8)
Headaches	8 (22.2)
Convulsions	2 (5.5)
Conjunctivitis	23 (63.8)
Myalgias	3 (8.3)
Lethargy	6 (16.6)

**Table 4:** Laboratory results at admission and discharge.

Laboratory Measures	Normal Range	Mean Values at Admission (Range)	Mean Values at Discharge (Range)
Hemoglobin, g/dL	11–16	11.36 (4.8–14.3)	12.65 (8–15.8)
White blood cells, 10^9^/L	4–12	11.29 (3.86–26.7)	11.61 (16.1–24.3)
Neutrophilia, n (%)		30 (83.3)	17 (47.2)
Lymphopenia, n (%)		32 (88.8)	18 (50)
Platelet count, 10^9^/L	150–400	254.45 (77–865)	534.49 (168–1077)
ESR, mm/h	5–12	57.35 (15–130)	26.9 (5–70)
C-reactive protein, mg/dL	<0.5	17.28 (0.8–37.1)	1.21 (0.08–8.8)
Ferritin, μg/L	11–124	696.73 (63.7–2435.9)	-
Procalcitonin, ng/mL	<0.5	4.4 (0.07–32)	-
Fibrinogen, mg/dL	150–400	823.83 (273.5–9710.6)	293 (201.1–491.4)
D-dimers, ng/mL	<400	2163.3 (575–5000)	-
ALT, U/L	0–50	44.56 (12–155)	50.15 (9–243)
AST, U/L	0–50	45.44 (8–279)	29.3 (4–75)
CK, U/L	0–150	86.91 (35–419)	17.08 (8–26)
Serum protein, g/dL	5.5–7.5	6.51 (5.3–7.9)	7.02 (6.3–7.7)
Albumin, g/L	3.5–5	3.58 (2.7–4.1)	-
Amylase, U/L	20–80	40.04 (18–89)	110.6 (27–291)
GGT, U/L	1–40	52.26 (6–214)	75.29 (13–257)
Glycemia, mg/dL	60–100	108.56 (63–171)	117.67 (78–188)
Serum iron, ug/dL	50–120	20.91 (4–61)	95.57 (31–186)
Creatinine, mg/dL	0.25–0.75	0.53 (0.11–1.21)	0.40 (0.18–0.65)
BUN, mg/dL	10–38	32.88 (16–116)	37 (15–58)
Proteinuria, n (%)		19 (52.77)	-
aPTT, s	24–36	28.54 (15.8–36.9)	23.79 (17.2–34.1)
PT, s	11–14	15.7 (11.9–21.7)	13.3 (11.7–16.1)
INR	0.8–1.3	1.36 (1.02–1.67)	1.24 (1.02–1.9)
LDH, U/L	180–430	309.79 (57–1008)	224.89 (133–411)
Triglycerides, mg/dL	0–150	176 (50–409)	279.6 (114–595)
Total lipids, mg/dL	400–800	625 (401–1080)	869.36 (492–1457)
Total cholesterol, mg/dL	0–200	164.9 (90–306)	214.67 (128–343)
Sodium, mmol/L	135–145	133.8 (127–139)	133.5 (129–138)
Potassium, mmol/L	3.5–5.2	4.05 (3.21–5.13)	4.72 (3.77–5.51)
Chlorine, mmol/L	100–110	101.21 (96–109)	98.5 (92–104)
Ionized calcium, mg/dL	4.2–5.2	4.11 (3.78–4.5)	4 (3.68–4.32)
Serum calcium, mg/dL	9–11	8.7 (7.8–9.4)	9 (8.2–9.9)
Magnesium, mg/dL	1.9–2.5	2.06 (1.68–2.81)	2.36 (1.84–2.8)
Phosphate, mg/dL	4–7	3.63 (2.23–4.51)	3.46 (2.71–4.62)
25-OH-Vitamin D, ng/mL	30–100	34.16 (15.5–89.95)	-

**Table 5:** Cardiac dysfunction.

Cardiac Dysfunction Markers	Values
CK-MB, ng/mL (range)	1.9 (<1–1.9)
Troponin I, ng/mL (range)	0.2 (0.05–1.45)
Myoglobin, ng/mL (range)	68.7 (21.9–126)
Brain natriuretic peptide (BNP), pg/mL (range)	243.63 (0.3–1210)
Echocardiograms performed, n (%)	33 (91.66)
LVEF median, % (range)	54; (35–65)
Left ventricle systolic dysfunction	9 (25)
LV diastolic dysfunction, n (%)	0
Any coronary abnormality, n (%)	2 (5.5)
LAD z score, median (range)	−0.02 (−1.3; 4)
RCA z score, median (range)	−0.5 (−1.1; 1.43)
Pericardial effusion, n (%)	5 (13.8)

**Table 6:** Imaging studies of organs affected by MIS-C.

Image Findings	Proportion of Patients Affected n (%)
Chest radiographies	30 (83.3)
Altered	14 (47)
Diffuse interstitial infiltrate	7 (23)
Pleural effusion	1 (3)
Interstitial infiltrate, localized	6 (20)
Atelectasis	1 (3)
Chest computed tomography	5 (14)
Pleural effusion	2 (40)
Ground-glass opacities	2 (40)
Pulmonary emphysema	1 (20)
Abdominal ultrasound	30 (83.3)
Hepatomegaly	13 (43.3)
Echogenic liver abnormalities	20 (66.6)
Biliary stasis	4 (13.3)
Splenomegaly	4 (13.3)
Echogenic kidneys abnormalities	11 (36.6)
Pyelocaliceal dilation	4 (13.3)
Renal corticomedullary differentiation loss	10 (33.3)
Fluid effusion	(30)

**Table 7:** Longitudinal analysis of clinical, laboratory parameters, and complications in patients with MIS-C at follow-up visits.

Variable	2 Weeks	1 Month	3 Months	6 Months	9 Months	12 Months
Patients, n	11	24	31	20	12	13
Females, n (%)	5 (45.4)	11	15 (48.3)	7 (35)	5 (41.7)	7 (53.8)
Males, n (%)	6 (54.5)	13	16 (51.6)	13 (65)	7 (58.3)	6 (446.2)
Age, mean	12.9	9.15	9.66	10.45	11	9.8
Positive IgG antibodies, n/N *	-	10/15	9/18	4/7	-	-
Inflammatory syndrome, n (%)	0	0	0	0	0	0
CRP mg/dL, mean	0.07	0.08	0.07	0.1	0.25	0.04
Anemia, n (%)	1 (9.09)	2 (8.3)	4 (12.8)	5 (25)	1 (8.3)	0
Liver cytolysis, n (%)	0	1 (4.16)	1 (3.2)	0	0	0
Coagulopathy, n (%)	0	0	1 (3.2)	0	3 (25)	0
Dyslipidemia, n (%)	8 (72.7)	13 (54.16)	4 (12.9)	0	0	0
Electrocardiogram modifications	0	2 (8.3)	5 (16.2)	1 (5)	0	0
Echocardiography, n (%)	4 (36.3)	12 (50)	8 (25.8)	3 (15)	0	0
LVEF (%), mean	60	63	64	53	-	-
Ventricular dysfunction	1	1	0	0	0	0
Coronary aneurysms or dilatations	1	1	1	0	0	0
Radiography or CT scan, n (%)	4 (36.3)	17 (47)	1 (3.2)	1 (5)	0	0
Lung sequelae	1 (9.09)	3 (12.5)	1 (3.2)	1	1	1
Abdominal ultrasound, n (%)	5 (45.4)	13 (54.1)	15 (48.3)	8 (40)	4 (33.3)	4 (30.7)
Liver echogenicity abnormalities, n (%)	3 (27.2)	8 (33.3)	4 (12.9)	2 (10)	0	1 (7.6)
Renal echogenicity	0	0	0	0	0	0

* N represents the total number of patients that were tested for SARS-CoV-2 antibodies.

## Data Availability

The original contributions presented in the study are included in the article, further inquiries can be directed to the corresponding author.

## Abbreviations

ALT	alanine transaminase
aPTT	activated partial thromboplastin time
AST	aspartate aminotransferase
BNP	brain natriuretic peptide
BUN	blood urea nitrogen
CDC	Centers for Disease Control and Prevention
CK-MB	creatine kinase-myoglobin binding
CK	creatine kinase
COVID-19	coronavirus disease 2019
CRP	C-reactive protein
ESR	erythrocyte sedimentation rate
GGT	gamma-glutamyl transferase
IgG	immunoglobulin G
IgM	immunoglobulin M
INR	international normalized ratio
IVIg	Intravenous Immunoglobulin
LAD	left anterior descending artery
LBBB	left bundle branch block
LDH	lactate dehydrogenase
LV	left ventricle
LVEF	left ventricle ejection fraction
MIS-C	Multisystemic Inflammatory syndrome in children
NS	not specified
PCR	polymerase chain reaction
PT	prothrombin time
RCA	right coronary artery
RCPCH	Royal College of Paediatrics and Child Health
SARS-CoV-2	severe acute respiratory syndrome coronavirus 2
WHO	World Health Organization

## References

[1] Royal College of Paediatrics and Child Health (2020). Guidance—Paediatric Multisystem Inflammatory Syndrome Temporally Associated with COVID-19 (PIMS). https://www.rcpch.ac.uk/resources/guidance-paediatric-multisystem-inflammatory-syndrome-temporally-associated-covid-19-pims.

[2] Centers for Disease Control and Prevention (2020). Multisystem Inflammatory Syndrome in Children (MIS-C) Associated with COVID-19 Summary and Recommendations. https://ndc.services.cdc.gov/case-definitions/multisystem-inflammatory-syndrome-in-children-mis-c-2023/.

[3] World Health Organization (2020). Multisystem Inflammatory Syndrome in Children and Adolescents Temporally Related to COVID-19—Scientific Brief. https://www.who.int/publications/i/item/multisystem-inflammatory-syndrome-in-children-and-adolescents-with-covid-19.

[4] Pouletty M., Borocco C., Ouldali N., Caseris M., Basmaci R., Lachaume N., Bensaid P., Pichard S., Kouider H., Morelle G. (2020). Paediatric multisystem inflammatory syndrome temporally associated with SARS-CoV-2 mimicking Kawasaki disease (Kawa-COVID-19): A multicentre cohort. Ann. Rheum. Dis..

[5] Kıymet E., Böncüoğlu E., Şahinkaya Ş., Cem E., Çelebi M.Y., Düzgöl M., Kara A.A., Arıkan K.Ö., Vuran G.T., Yılmazer M.M. (2021). A Comparative study of children with MIS-C between admitted to the pediatric intensive care unit and pediatric ward: A one-year retrospective study. J. Trop. Pediatr..

[6] Davies P., Evans C., Kanthimathinathan H.K., Lillie J., Brierley J., Waters G., Johnson M., Griffiths B., du Pré P., Mohammad Z. (2020). Intensive care admissions of children with paediatric inflammatory multisystem syndrome temporally associated with SARS-CoV-2 (PIMS-TS) in the UK: A multicentre observational study. Lancet Child Adolesc. Health.

[7] Belot A., Antona D., Renolleau S., Javouhey E., Hentgen V., Angoulvant F., Delacourt C., Iriart X., Ovaert C., Bader-Meunier B. (2020). SARS-CoV-2-related paediatric inflammatory multisystem syndrome, an epidemiological study, France, 1 March to 17 May 2020. Eurosurveillance.

[8] Godfred-Cato S. (2020). COVID-19–Associated multisystem inflammatory syndrome in children—United States, March–July 2020. Mmwr-Morbidity Mortal. Wkly. Rep..

[9] Hoste L., Van Paemel R., Haerynck F. (2021). Multisystem inflammatory syndrome in children related to COVID-19: A systematic review. Eur. J. Pediatr..

[10] Jiang L., Tang K., Levin M., Irfan O., Morris S.K., Wilson K., Klein J.D., Bhutta Z.A. (2020). COVID-19 and multisystem inflammatory syndrome in children and adolescents. Lancet Infect. Dis..

[11] Dionne A., Son M.B.F., Randolph A.G. (2022). An Update on Multisystem Inflammatory Syndrome in Children Related to SARS-CoV-2. Pediatr. Infect. Dis. J..

[12] Jenke A., Steinmetz M. (2023). Paediatric Inflammatory Multisystem Syndrome—Temporally Associated with SARS-CoV-2 (PIMS-TS)—A German Single Centre Real-Life Evaluation of the Swiss and UK Consensus Statements. Cardiol. Young.

[13] Flood J., Shingleton J., Bennett E., Walker B., Amin-Chowdhury Z., Oligbu G., Avis J., Lynn R.M., Davis P., Bharucha T. (2021). Paediatric Multisystem Inflammatory Syndrome Temporally Associated with SARS-CoV-2 (PIMS-TS): Prospective, National Surveillance, United Kingdom and Ireland, 2020. Lancet Reg. Health-Eur..

[14] Abrams J.Y., E Oster M., E Godfred-Cato S., Bryant B., Datta S.D., Campbell A.P., Leung J.W., A Tsang C., Pierce T.J., Kennedy J.L. (2021). Factors Linked to Severe Outcomes in Multisystem Inflammatory Syndrome in Children (MIS-C) in the USA: A Retrospective Surveillance Study. Lancet Child Adolesc. Health.

[15] Duong-Quy S., Huynh-Truong-Anh D., Le-Thi-Hong N., Le-Van T., Le-Thi-Kim S., Nguyen-Quang T., Nguyen-Thi-Kim T., Nguyen-Phuong N., Nguyen-Chi T., Nguyen-Van T. (2022). Acute Respiratory Distress Syndrome Associated with Multisystem Inflammatory Syndrome in a Child with COVID-19 and Diabetic Ketoacidosis: A Case Report. Pulm. Ther..

[16] Valverde I., Singh Y., Sanchez-De-Toledo J., Theocharis P., Chikermane A., Di Filippo S., Kucinska B., Mannarino S., Tamariz-Martel A., Gutierrez-Larraya F. (2021). Acute Cardiovascular Manifestations in 286 Children with Multisystem Inflammatory Syndrome Associated with COVID-19 Infection in Europe. Circulation.

[17] Vieira A.P.R., Carvalho P.R.A., Machado S.H., da Rocha T.S. (2025). Clinical and Laboratory Markers Defining MIS-C and Hyperinflammation in COVID-19: A Cross-Sectional Study in a Tertiary Hospital. Hortic. Bras..

[18] Alsaied T., Tremoulet A.H., Burns J.C., Saidi A., Dionne A., Lang S.M., Newburger J.W., de Ferranti S., Friedman K.G. (2021). Review of Cardiac Involvement in Multisystem Inflammatory Syndrome in Children. Circulation.

[19] Young T.K., Shaw K.S., Shah J.K., Noor A., Alperin R.A., Ratner A.J., Orlow S.J., Betensky R.A., Shust G.F., Kahn P.J. (2021). Mucocutaneous Manifestations of Multisystem Inflammatory Syndrome in Children During the COVID-19 Pandemic. JAMA Dermatol..

[20] Miller J., Cantor A., Zachariah P., Ahn D., Martinez M., Margolis K.G. (2020). Gastrointestinal Symptoms as a Major Presentation Component of a Novel Multisystem Inflammatory Syndrome in Children That Is Related to Coronavirus Disease 2019: A Single Center Experience of 44 Cases. Gastroenterology.

[21] Tullie L., Ford K., Bisharat M., Watson T., Thakkar H., Mullassery D., Giuliani S., Blackburn S., Cross K., De Coppi P. (2020). Gastrointestinal Features in Children with COVID-19: An Observation of Varied Presentation in Eight Children. Lancet Child Adolesc. Health.

[22] Sahn B., Eze O.P., Edelman M.C., Chougar C.E., Thomas R.M., Schleien C.L.M., Weinstein T. (2021). Features of Intestinal Disease Associated with COVID-Related Multisystem Inflammatory Syndrome in Children. J. Pediatr. Gastroenterol. Nutr..

[23] Faust S.N., Haynes R., E Jones C., Staplin N., Whittaker E., Jaki T., Juszczak E., Spata E., Wan M., Bamford A. (2024). Immunomodulatory Therapy in Children with Paediatric Inflammatory Multisystem Syndrome Temporally Associated with SARS-CoV-2 (PIMS-TS, MIS-C; RECOVERY): A Randomised, Controlled, Open-Label, Platform Trial. Lancet Child Adolesc. Health.

[24] Varadarajan P., Solomon R.S., Subramani S., Subramanian R., Srividya G., Raghunathan E. (2025). Cardiovascular Involvement in Multisystem Inflammatory Syndrome in Children and Midterm Follow-Up from a Pediatric Tertiary Center in India. World J. Clin. Pediatr..

[25] Dionne A., Mah D.Y., Son M.B.F., Lee P.Y., Henderson L., Baker A.L., de Ferranti S.D., Fulton D.R., Newburger J.W., Friedman K.G. (2020). Atrioventricular Block in Children with Multisystem Inflammatory Syndrome. Pediatrics.

[26] Sperotto F., Friedman K.G., Son M.B.F., VanderPluym C.J., Newburger J.W., Dionne A. (2021). Cardiac Manifestations in SARS-CoV-2-Associated Multisystem Inflammatory Syndrome in Children: A Comprehensive Review and Proposed Clinical Approach. Eur. J. Pediatr..

[27] Hejazi O.I., Loke Y.-H., Harahsheh A.S. (2021). Short-term Cardiovascular Complications of Multi-system Inflammatory Syndrome in Children (MIS-C) in Adolescents and Children. Curr. Pediatr. Rep..

[28] Matsubara D., Kauffman H.L., Wang Y., Calderon-Anyosa R., Nadaraj S., Elias M.D., White T.J., Torowicz D.L., Yubbu P., Giglia T.M. (2020). Echocardiographic Findings in Pediatric Multisystem Inflammatory Syndrome Associated with COVID-19 in the United States. J. Am. Coll. Cardiol..

[29] Theocharis P., Wong J., Pushparajah K., Mathur S.K., Simpson J.M., Pascall E., Cleary A., Stewart K., Adhvaryu K., Savis A. (2021). Multimodality Cardiac Evaluation in Children and Young Adults with Multisystem Inflammation Associated with COVID-19. Eur. Heart J.-Cardiovasc. Imaging.

[30] Winant A.J., Blumfield E., Liszewski M.C., Kurian J., Foust A.M., Lee E.Y. (2020). Thoracic Imaging Findings of Multisystem Inflammatory Syndrome in Children Associated with COVID-19: What Radiologists Need to Know Now. Radiol. Cardiothorac. Imaging.

[31] Rostad B.S., Shah J.H., Rostad C.A., Jaggi P., Richer E.J., Linam L.E., Alazraki A.L., Riedesel E.L., Milla S.S. (2021). Chest Radiograph Features of Multisystem Inflammatory Syndrome in Children (MIS-C) Compared to Pediatric COVID-19. Pediatr. Radiol..

[32] Feldstein L.R., Tenforde M.W., Friedman K.G., Newhams M., Rose E.B., Dapul H., Soma V.L., Maddux A.B., Mourani P.M., Bowens C. (2021). Characteristics and Outcomes of U.S. Children and Adolescents with Multisystem Inflammatory Syndrome in Children (MIS-C) Compared with Severe Acute COVID-19. JAMA.

[33] Riphagen S., Gomez X., Gonzalez-Martinez C., Wilkinson N., Theocharis P. (2020). Hyperinflammatory Shock in Children During COVID-19 Pandemic. Lancet.

[34] Verdoni L., Mazza A., Gervasoni A., Martelli L., Ruggeri M., Ciuffreda M., Bonanomi E., D’ANtiga L. (2020). An Outbreak of Severe Kawasaki-Like Disease at the Italian Epicentre of the SARS-CoV-2 Epidemic: An Observational Cohort Study. Lancet.

[35] Feldstein L.R., Rose E.B., Horwitz S.M., Collins J.P., Newhams M.M., Son M.B.F., Newburger J.W., Kleinman L.C., Heidemann S.M., Martin A.A. (2020). Multisystem Inflammatory Syndrome in U.S. Children and Adolescents. N. Engl. J. Med..

[36] Cantor A., Miller J., Zachariah P., DaSilva B., Margolis K., Martinez M. (2020). Acute Hepatitis Is a Prominent Presentation of the Multisystem Inflammatory Syndrome in Children: A Single-Center Report. Hepatology.

[37] Giannattasio A., Maglione M., D’anna C., Muzzica S., Pappacoda S., Lenta S., Di Mita O., Ranucci G., Mandato C., Tipo V. (2022). Liver and Pancreatic Involvement in Children with Multisystem Inflammatory Syndrome Related to SARS-CoV-2: A Monocentric Study. Children.

[38] Al-Ghafry M., Vagrecha A., Malik M., Levine C., Uster E., Aygun B., Appiah-Kubi A., Vlachos A., Capone C.A., Rajan S. (2021). Multisystem Inflammatory Syndrome in Children (MIS-C) and the Prothrombotic State: Coagulation Profiles and Rotational Thromboelastometry in a MIS-C Cohort. J. Thromb. Haemost..

[39] Trapani S., Rubino C., Lasagni D., Pegoraro F., Resti M., Simonini G., Indolfi G. (2022). Thromboembolic Complications in Children with COVID-19 and MIS-C: A Narrative Review. Front. Pediatr..

[40] El-Halaby H., Eid R., Elagamy A., El-Hussiny A., Moustafa F., Hammad A., Zeid M. (2024). A Retrospective Analysis of Acute Kidney Injury in Children with Post-COVID-19 Multisystem Inflammatory Syndrome: Insights into Promising Outcomes. Ital. J. Pediatr..

[41] Lipton M., Mahajan R.G., Kavanagh C., Shen C.L., Batal I., Dogra S., Jain N.G., Lin F., Uy N.S. (2021). AKI in COVID-19–Associated Multisystem Inflammatory Syndrome in Children (MIS-C). Kidney360.

[42] Levin M. (2020). Childhood Multisystem Inflammatory Syndrome—A New Challenge in the Pandemic. N. Engl. J. Med..

[43] Baradaran A., Malek A., Moazzen N., Shaye Z.A. (2020). COVID-19 Associated Multisystem Inflammatory Syndrome: A Systematic Review and Meta-Analysis. Iran. J. Allergy Asthma Immunol..

[44] Shyong O., Alfakhri N., Bates S.V., Carroll R.W., Gallagher K., Huang L., Madhavan V., Murphy S.A., Okrzesik S.A., Yager P.H. (2025). Multisystem Inflammatory Syndrome in Children: A Comprehensive Review Over the Past Five Years. J. Intensive Care Med..

[45] Ahmed M., Advani S., Moreira A., Zoretic S., Martinez J., Chorath K., Acosta S., Naqvi R., Burmeister-Morton F., Burmeister F. (2020). Multisystem Inflammatory Syndrome in Children: A Systematic Review. EClinicalMedicine.

[46] Haslak F., Barut K., Durak C., Aliyeva A., Yildiz M., Guliyeva V., Varol S.E., Cebeci S.O., Aygun F., Varli Y.Z. (2021). Clinical Features and Outcomes of 76 Patients with COVID-19-Related Multisystem Inflammatory Syndrome in Children. Clin. Rheumatol..

[47] Davies P., du Pré P., Lillie J., Kanthimathinathan H.K. (2021). One-Year Outcomes of Critical Care Patients Post–COVID-19 Multisystem Inflammatory Syndrome in Children. JAMA Pediatr..

[48] Syrimi E., Fennell E., Richter A., Vrljicak P., Stark R., Ott S., Murray P.G., Al-Abadi E., Chikermane A., Dawson P. (2021). The Immune Landscape of SARS-CoV-2-Associated Multisystem Inflammatory Syndrome in Children (MIS-C) from Acute Disease to Recovery. iScience.

[49] D’aUria E., Bova S.M., Dallapiccola A.R., De Santis R., Leone A., Calcaterra V., Mannarino S., Garbin M., Olivotto S., Zirpoli S. (2024). Long-Term Health Outcome and Quality of Life in Children with Multisystem Inflammatory Syndrome: Findings from Multidisciplinary Follow-Up at an Italian Tertiary-Care Paediatric Hospital. Eur. J. Pediatr..

[50] Glazyrina A., Zholobova E., Iakovleva E., Bobkova P., Krasnaya E., Kovygina K., Romanova O., Blyuss O., Tutelman K., Petrova P. (2024). Short-Term and Medium-Term Clinical Outcomes of Multisystem Inflammatory Syndrome in Children: A Prospective Observational Cohort Study. Ital. J. Pediatr..

[51] Capone C.A., Misra N., Ganigara M., Epstein S., Rajan S., Acharya S.S., Hayes D.A., Kearney M.B., Romano A., Friedman R.A. (2021). Six Month Follow-Up of Patients with Multi-System Inflammatory Syndrome in Children. Pediatrics.

[52] Penner J., Abdel-Mannan O., Grant K., Maillard S., Kucera F., Hassell J., Eyre M., Berger Z., Hacohen Y., Moshal K. (2021). 6-Month Multidisciplinary Follow-Up and Outcomes of Patients with Paediatric Inflammatory Multisystem Syndrome (PIMS-TS) at a UK Tertiary Paediatric Hospital: A Retrospective Cohort Study. Lancet Child Adolesc. Health.

